# Improvement of Heliciculture by Three Medicinal Plants Belonging to the Lamiaceae Family

**DOI:** 10.1155/2019/2630537

**Published:** 2019-10-21

**Authors:** Lamiaa Lemjallad, Rachida Chabir, Youssef Kandri Rodi, Lahssen El Ghadraoui, Fouad Ouazzani Chahdi, Faouzi Errachidi

**Affiliations:** ^1^Department of Chemistry, Laboratory of Applied Organic Chemistry, Faculty of Sciences and Technologies, University Sidi Mohamed Ben Abdellah, B.P. 2202-route Imouzzer, Fez, Morocco; ^2^Team of Nutrition, Agri-Food and Environment, Laboratory of Human Pathology, Biomedicine and Environment, Faculty of Medicine and Pharmacy, University Sidi Mohamed Ben Abdellah, P.B. 1893 km 2200 Road Sidi Harazem, Fez, Morocco; ^3^Laboratory of Functional Ecology and Environment, Faculty of Sciences and Technologies, University Sidi Mohamed Ben Abdellah, B.P. 2202-route Imouzzer, Fez, Morocco

## Abstract

Snails were fed with three medicinal plants belonging to the Lamiaceae family (rosemary, sage, and peppermint) in order to test their effects on those animals with high nutritive values. The media of raising were flour containing different percentages of the cited plants ranging from 1% to 9%. The feed had benefits on the raised snails depending on the plant and its percentage. Minerals in those aromatic plants, especially zinc and magnesium, had their effect on protein synthesis in snails fed with those plant percentages. Rosemary was the most profitable plant with the highest protein amount, the lowest mortality rate, and reduced microbial charge. Furthermore, it was a good regulator of the specific catalase activity which confirmed the role of the antioxidant activity of rosemary during raising snails.

## 1. Introduction

Lamiaceae is a cosmopolitan botanical family, it includes 186 genera with a total of 7200 spices all over the world [1] and 200 species grouped into 28 genera in Morocco [2]. This family possesses high phenolic compound content such as polyphenols, tannins, iridoids, quinones, coumarins, diterpenoids, triterpenoids, saponins, and in some cases, pyridine and pyrrolidine alkaloids [2]. This diversity of bioactive molecules enables this family to have a positive impact on human health, such as antioxidant [3], antimicrobial [4], insecticidal, and acaricidal activities [5]. Also, they prevent aging phenomena and several chronic diseases in humans [6]. The Lamiaceae family is well known for its specific phenolic compounds such as rosmarinic acid, carvacrol, and thymol, occurring in several genera of the Lamiaceae family which are responsible for their antioxidant properties [7]. Actually, the use of the natural phenolic compounds having antioxidant activity is much better and safer than using the synthetic ones [8].

The decrease in phenolic compounds in biological systems cause a diminution in antioxidant defenses correlated with the increase of reactive oxygen species (ROS) causing oxidative stress [9] responsible for some pathologies development such as obesity, diabetes type 2, atherosclerosis, cardiovascular [10], schizophrenia [11], cancer, Alzheimer's, and Parkinson's diseases [9]. These ROS have the ability to oxidize a number of biological molecules such as carbohydrates, nucleic acids, fats, and proteins [12]. Oxidation is also the second origin of food degradation causing a real damaging in it which is reflected in a negative effect on nutritional and sensory qualities [6].

Besides phenolic compounds, minerals have human health benefits. Minerals are fundamentally metals and other inorganic compounds constituting about 4 to 6 percent of body weight. They are indispensable for normal nutrition, and it has been confirmed that they have an important role than vitamins because they are required for vitamin absorption. Minerals are responsible for bone and teeth hardness, play an essential function in metabolism, are components of enzymes systems, and constitute many organic molecules [13].

To assess the aromatic plants' effects on nutritional values and oxidative stress impact in vivo, we have chosen snails as an animal model. The latter have the ability to evaluate the environmental impact of heavy metals [14], and they can accumulate different classes of chemicals such as pesticides and xenobiotics [15]. On the other hand, snails are an intermediate host for microorganisms causing diseases for human, domesticated animals, and wildlife [16]. A study by Brockelman and Jackson affirm that 90% of Moroccan snails were microbiologically infected [17]. In addition, many studies conducted on *Helix aspersa* and their contamination by pathogen bacteria such as *Salmonella*. It was isolated from 43% of Moroccan *Helix aspersa* contaminating their flesh [18].

The snail *Helix aspersa*, the organism used in this study, belongs to the Helicidae family of snails and has a high distribution rate all over the world. Besides, it is the most common land snails in the world, it presents food of high nutritive value with a shell mainly composed of calcium carbonate, a flesh consisting of water (at least 70%) and protein (about 60–70% dry basis analysis) [19]. Unfortunately, the Moroccan population does not benefit from this precious nutritive source caused by their unawareness of snails' positive effects on health. Besides that, land snails are not present in the Moroccan diet, and their consumption is limited in one traditional preparation. This work targets the evaluation of the nutritional value, the microbial infection, and the oxidative stress in *Helix aspersa* raised in the presence of aromatic plant powders from the Lamiaceae family.

## 2. Materials and Methods

### 2.1. Raw Material and Chemicals

Medicinal plants used in this study were peppermint (*Mentha piperita*), sage (*Salvia officinalis*), and rosemary (*Rosmarinus officinalis*) cultivated in Fes, Morocco.

### 2.2. Heavy Metal in Plants

Samples were grounded for heavy metal estimation. They were digested in the mixture of nitric acid and perchloric acid (HNO_3_ : HClO_4_; 10 : 4, v/v). The digested samples were then filtered, and the content of mineral elements, such as Al, Ba, Ca, Fe, K, Mg, Mn, Na, P, Sr, and Zn, were determined by inductively coupled plasma optical emissions spectrometry (ICP-AES) [20].

### 2.3. Snail Raising

The snails used in this study were taken from a dormancy phase to align their physiological state. To initiate the experiment, snails were sprayed with sterilized physiological water in order to induce their dormancy lifting. The experimental trial was started once the snails emerged from their shell.

Snails (*Helix aspersa*) used had an average weight of 0.95 g (an average size of 15.5 mm) and were placed in plastic boxes (size 15 × 30 × 25 cm) with a perforated lid (Ø = 3 mm). They were fed daily with 15 grams of flour containing 0%, 1%, 3%, 6%, and 9% of powdered medicinal plants (peppermint, sage, and rosemary). The raising system was humidified (80%) daily to ensure optimum growth in culture chamber at 26°C. Daily monitoring was carried out to estimate weight and morphometric quantifications for a period of 9 days, which is the critical period where we notice the highest mortality rate in raising stations.

### 2.4. Catalase Assay

Catalase assay was carried out by the colorimetric method (Sinha [21]) using the dichromate/acetic acid reagent. The latter was prepared by mixing 5% K_2_Cr_2_O_7_ aqueous solution with acetic acid (1 : 3; v : v). The assay was performed by adding 100 *μ*l of the snail extract (crushed and centrifuged in the phosphate buffer) with 300 *μ*l of H_2_O_2_ (2 mM) and the phosphate buffer solution (pH = 7.4; 20 mM). After 5 min, 2 ml of the dichromate/acetic acid reagent was added to stop the reaction. After 10 min, evaluation was carried out at 570 nm, and a calibration curve for hydrogen peroxide (H_2_O_2_) was run in order to calculate the enzymatic units [21].

### 2.5. Protein Dosage

Snail protein extracts were evaluated by using the modified Biuret method. The Gornall reagent was prepared with 0.15% copper sulphate (CuSO_4_ 5 H_2_O), 0.6% potassium sodium tartrate (KNaC_4_H_4_O_6_·6H_2_O), 3% sodium hydroxide (NaOH), and 0.1% potassium iodide (KI). 4 ml of this reagent is added to 1 ml of the diluted crushed snail extract. The mixture is left in the dark for 30 minutes before reading the absorbance in a spectrophotometer at 540 nm [22].

### 2.6. Microbiological Counting

Microbiological counting was done in order to quantify microorganisms in untreated snails (Template) and those fed with powdered aromatic plants in flour. It was evaluated by placing 100 *μ*l of crushed snail dilution on different culture media (King B, Brain Heart Infusion Broth, Malt, and YPG (1% yeast extract, 2% peptone, and 2% glucose)) targeting microbial strains that may be related to snail mortality rates. Petri dishes containing culture media were incubated (37°C for bacteria and 25°C for yeasts and molds). The qualitative and quantitative analysis of the results was carried out after 24 hours for the bacteria and 48 hours for the yeasts.

## 3. Results and Discussion

### 3.1. Mineral Content in Plants

According to Table 1, we noticed that rosemary have the least mineral content followed by sage and peppermint. The macrominerals, calcium (Ca), potassium (K), sodium (Na), and phosphorus (P), had their highest values in the powdered peppermint. On the other hand, magnesium (Mg) had the highest content in the powdered sage. Calcium is an essential mineral in human health; it is an essential compound playing vital roles in having strong bones and teeth, blood clotting, fertilization, stabilizes many proteins, and nerve impulse conduction [23]. Magnesium is needed for the activation of over 300 enzymes, energy formation and exchange, glucose transportation between membranes, proper activity of insulin, and all reactions involving phosphorylation. Also, it influences oxidative stress, inflammation, immune function, and neurological and cardiovascular functions [24]. Zinc had the highest value in powdered rosemary, and it plays an important role because it constitutes many vital enzymes, modulates neurotransmission, and involves in protein synthesis, DNA regulation, and protein-protein interactions [25]. Manganese is an important element for human growth, development, and maintenance of health. It is needed for a variety metabolic functions such as energy metabolism, activation of certain metalloenzymes, nervous system function, immunological system function, reproductive hormone function, blood clotting, brain development, and in antioxidant enzymes that protect cells from damage due to free radicals [26].

### 3.2. Snail Raising

**Figure 1:**
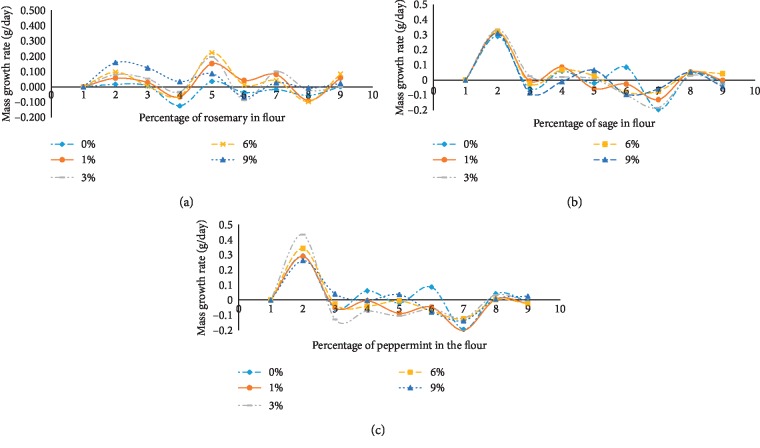
Snails mass growth rate in function of time in the presence of rosemary (a), sage (b), and peppermint (c) powder in flour.

The medicinal plants tested during the land snail farming were rosemary, sage, and peppermint. Each plant has its influence on the evolution and the weight gain during the raising, and the growth rate is shown in Figure 1. A remarkable weight gain in the second day of snail husbandry was noted, and this probably was due to their high hydration after dormancy lifting. Beyond that, there was a progressive decrease in snail weight feeding with the various percentages of peppermint powder (Figure 1(c)) in contrast to control snails (flour without peppermint). So, we concluded that peppermint was not a suitable plant for raising snails. According to Figures 1(b) and 1(c), we noticed that the snail growth rate during the time was limited between −0.005 and 0.005 (g/day) which confirmed their slow or stopped growth. When we add 6% of sage powder in the snail feed, we noticed that the growth rate was regular confirming that there was not any loss of weight.

For rosemary (Figure 1(a)), we noticed a significant weight gain in all the land snails fed with the various percentages of powdered rosemary biomass in flour from 0 to 9%. That was shown by the average of the growth rate limited between −3 and 5 (g/day).

*Helix aspersa* is largely used in laboratory as a biological monitor for heavy metal toxicity. A study conducted by Simkiss and his collaborators has shown that adding antibiotic (tetracycline and nalidixic acid) to *Helix aspersa* diet did not affect the food consumption in contrast to zinc nitrate which reduced feeding to 38%. This study showed that the feeding rate did not affect the weight gain of these little organisms even by the use of zinc nitrate or antibiotics [27]. This confirms that the medicinal plants have a positive impact on *Helix aspersa* digestive system.

**Figure 2:**
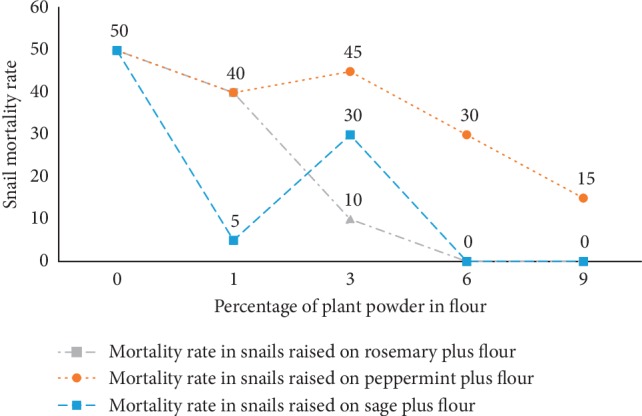
Snail mortality rate in function of the medicinal plant powder percentage added to raising medium.

Snail mortality was influenced by the feeding with flour containing medicinal plants powder. According to results obtained in Figure 2, we noticed a weak reduction in the snail mortality rate by adding the different percentages of peppermint powder in flour, and there was a remarkable snail mortality rate even in the medium containing the highest percentage of plant powder (9%) in their feed. In snails fed with sage and rosemary, we noticed a significant reduction in the mortality rate until a massive reduction at 6% of those powdered plants. Moreover, snails fed with powdered rosemary in flour had a mortality rate reduction, progressive and correlating with the increase in the rosemary biomass, until total snail mortality at 6% and 9% of powder in flour which means that it was the best snail protective and it adjusted their weight.

**Figure 3:**
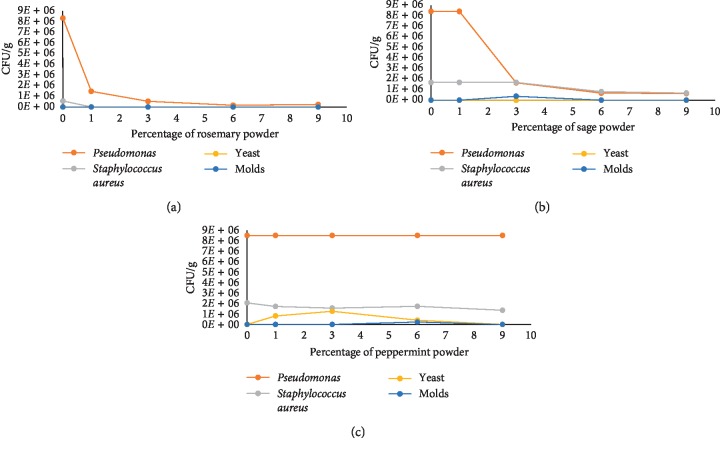
Microbial load of snail extracts (CFU/g) as percentages of the medicinal plant introduced rosemary (a), sage (b), and peppermint (c).

Mortality rate variation might be caused by the microbial charge shown in snails (Figure 3). A study conducted by Napoli and his collaborators confirms that the *Aelurostrongylus abstrusus* lungworm (*L*_1_) injection in the posterior-ventral portion of the *Helix aspersa* foot increases the mortality rate from 40% (mortality rate for uninfected) to 60% (infected snails by injection) [28]. There are other factors responsible for the mortality of the *H. aspersa* gastropod such as the toxicity by chemical compounds. Radwan and Mohamed demonstrate that the feeding of *H. aspersa* with different concentration of Imidacloprid causes a high mortality rate, and the mortality rate increases with the concentration added in the food used during the raising [14].

### 3.3. Protein Evolution in Raised Snails Biomass

**Figure 4:**
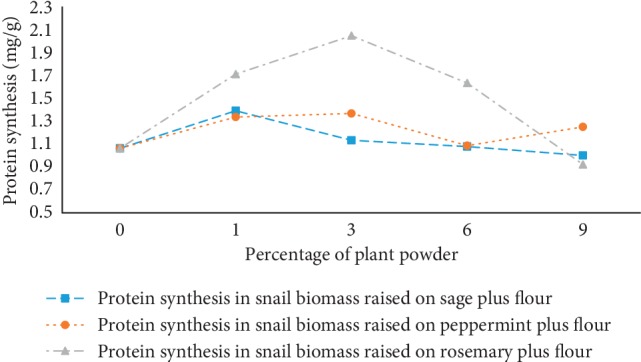
Protein synthesis in snails treated with medicinal plants.

The medicinal plants (sage, peppermint, and rosemary) affect the protein synthesis in snails (*Helix aspersa*) which varied from one plant to another and its powder quantity (%) included in the snail-raising medium. From Figure 4, it was found that the formulas 1% and 3% induced protein synthesis, whereas the 6% and 9% formula inhibit their synthesis. This confirms that the latter concentrations initiate toxicity on snails and explains the choice of the snail protein extract from the formula containing 6% as an optimal value to maximize antioxidant and antimicrobial activities (Figure 3). In addition, the treatment with rosemary induce a higher protein content and that might be due to the synthesis protein stress implicated in the oxidative stress and its reaction on cells as a response to this phenomenon. These results agree with those of Radwan and Mohamed [14] who found that the protein concentration increase with the Imidacloprid concentration (synthetic insecticide). This was caused by the imbalance between the rate synthesis and rate degradation of protein during the experiments [14].

The higher protein synthesis in snails fed with powdered rosemary in flour was explicated by the presence of good antioxidant reducing protein damage even in presence of several metals. It is known that antioxidant exhibit pro-oxidant effect in the presence of metals such as copper-producing protein damage. To study the effect of antioxidant in the presence of copper, Moreno et al. investigated the oxidative protein damage caused by methanol rosemary extract, ascorbate, and 6-hydroxy-2,5,7,8-tetramethylchroman-2-carboxylic acid (Trolox). The results show that 20 *μ*g of methanol rosemary extract induces reduced protein damage compared with 20 *μ*M of Trolox and ascorbate. Those results confirm that the presence of copper does not influence the protective effect of rosemary as an antioxidant contrary to synthetic antioxidant (Trolox and ascorbate) [29].

### 3.4. Catalase Activity in Snail Biomass

**Figure 5:**
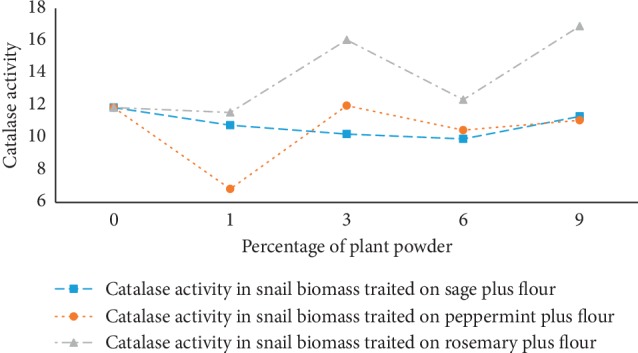
Catalase activity expressed as intracellular antioxidant activity in snails during farming with different percentages of powdered medicinal plants.

Catalase enzyme activity was introduced in this study to evaluate oxidative stress in the studied snails. Catalase activity in treated snails with medicinal plants varied according to the plant and its powder percentages in the snail-raising medium. From Figure 5, we notice that the highest catalase activity values were obtained in snails treated with rosemary. This was due to the modulation of intracellular oxidative stress evaluated in snails “*Helix aspersa*” during raising. Catalase activity has a high disorder at different powder percentages of peppermint, and this confirms that it was not a good regulator of catalase activity. For the sage, there was a small decrease in catalase activity on all the biomass percentages used in the study except in the 9% biomass powder where we noticed a slight increase. This reduction in snails' catalase activity fed in the presence of powdered sage was confirmed by a study in vitro by Upadhyay and his collaborators [30].

Several things can influence the oxidative stress in *Helix aspersa* and among them we find soil contamination with organic and inorganic pollutants causing an increase in the catalase activity responsible for cell damages [15]. Works done by Radwan and Mohamed where they use Imidacloprid (insecticide), it shows that catalase activity increased during the treatment from the first day to the seventh. This catalase activity was a result of oxygen-free radical accumulation causing oxidative stress and cell damage [14].

### 3.5. Specific Catalase Activity Evolution

**Figure 6:**
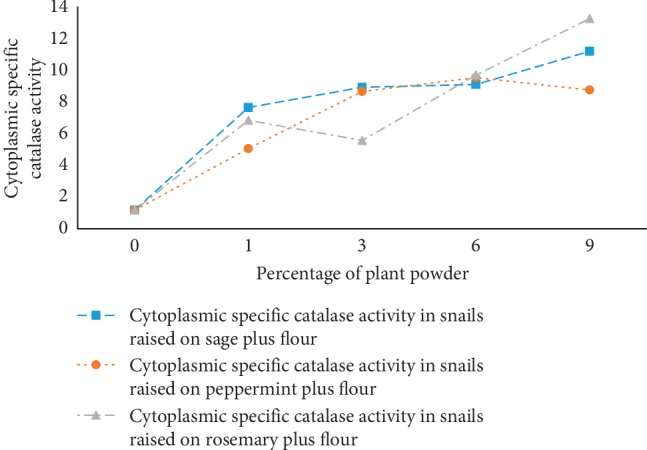
Specific catalase activity of “*Helix aspersa*” snails fed with powdered *Rosmarinus officinalis*, *Salvia officinalis*, and *Mentha piperita* at different percentages in flour.

Specific catalase activities were monitored as a function of the feed containing flour with percentages of powdered dry plant in the raising media. The results are shown in Figure 6. Formulas 1, 3, 6, and 9% induced specific catalase activity in a proportional manner. From this figure, we notice that the formulas integrating peppermint powder have a saturation in specific catalase activity from 3%, whereas there was no saturation with the other two plants (sage and rosemary). This shows that the 3 formulas induce catalase activity which is a physiological indicator of oxidative stress and cellular antioxidant activity.

### 3.6. Antimicrobial Activity

The presence of bacteria has been shown in a previous study, Serrano et al. confirm that 43% of uncooked Moroccan snails (*Helix aspersa*) are contaminated by *Salmonella*, and it occurs in shell and body and then penetrates into flesh [31]. Infections with salmonella cause clinical disorders including fever, abdominal pain, diarrhea, nausea, and vomiting. Also, it is responsible for numerous illness such pneumonia, meningitis, and osteomyelitis [32].

According to Figure 3(b), it was found that the usage of 3% of sage powder in livestock feed caused *Pseudomonas* sp. reduction with 6.10^5^ colony forming units (CFU/g) in snails. That allowed mold growth but with a very low number. The increase of mortality at this formula was explained by the fact that the *Staphylococcus* sp. and *Pseudomonas* sp. have a strong influence on snail mortality. *Pseudomonas* sp. and *Staphylococcus* sp. colonies were reduced in snail raising with 6% and 9% of sage powder, and those of molds were canceled, which reduced the mortality rate (Figure 2). In the case of peppermint (Figure 3(c)), we can notice that it didn't have any inhibitory effect on bacteria of the genus *Pseudomonas* sp. in snails at any percentage of powder feed plant. Despite this, at 1% and 3% of powder, there was a slight decrease in bacteria colony numbers of the genus *Staphylococcus* sp., which is responsible for decreased mortality rate even if there was a remarkable increase in yeasts. For the snail raising in media 6% and 9%, there was a decrease of colony numbers of the genus *Staphylococcus* sp., which influences the mortality rate. From Figure 3(a), it was found that the colony numbers of all strains decrease in snails with the increase in the percentage of rosemary in the experiments. Bacteria belonging to genus *Pseudomonas* sp. and *Staphylococcus* sp. were dropped at 1% of powdered rosemary introduced in the snail feed.

From those results, we can suggest that there was a correlation between the microorganism counting and the mortality rate of *H. aspersa*. As we can notice bacteria, yeast, and molds were inhibited by rosemary (Figure 2 (rate mortality)). The microbial charge diminution in the snails causes a mortality rate diminution.

In our study, we used the leaves grounded including essential oil, phenolic compounds, etc. On the other hand, essential oils were investigated for their antimicrobial activity in other studies. Sage and rosemary essential oils were studied for their antimicrobial activity in the conservation of fish fillets, and similar results were reported. Fish fillets immerged for 3 minutes in emulations of essential oil and stored for 24 days in a refrigerator (2°C). The results show that the fish fillets treated with rosemary essential oil delay the rate of psychrotrophic bacteria and *Enterobacteriacea* growth than those emerged in sage essential oil [33]. Also, it was demonstrated that rosemary and sage are natural antibacterial substances, and rosemary has the best antibacterial activity against food-associated microorganisms (against *Enterococcus*, *Bacillus*, and *Salmonella*) than sage [34].

Rosemary and sage had strong antimicrobial activity and had a role as antibiotics. Their antimicrobial effects on snails were compared with the effects of antibiotics such as tetracycline and nalidixic acid. Those antibiotics reduce the number of bacteria in alimentary tract which allows the intestines to be thinner than usual [27]. Most of the organisms present in the alimentary tract of *H. aspersa* are Gram-negative belonging to *Pseudomonas* sp., *Xanthomonas* sp., *Acinetobacter* sp., *Vibrio*, and the Enterobacteria. Some Gram-positive and endospore-forming bacteria of *Bacillus* spp. and species of *Staphylococcus* and *Micrococcus* were also present [35].

The antimicrobial activity of rosemary against *S. aureus* is investigated compared with natural preservative (benzoic acid) and synthetic ones such as butylhydroxytoluene (BHT) and butylated hydroxyanisole (BHA). Methanol rosemary extract has the highest antimicrobial activity against *S. aureus* followed by BHA, benzoic acid, and BHT [29].

## 4. Conclusion

The obtained results may change the herbs used in cooking snails and may integrate or modulate its composition. The results confirm that rosemary is a source of bioactive molecules responsible for its antimicrobial and antioxidant activity and influences the snail raising and make it profitable. By adding powdered rosemary to the raising media, we notice a decrease in the microbial charge in snails and especially *Pseudomonas* sp. and *Staphylococcus aureus*. In the case of sage, it was the second plant that positively influenced the snail raising, and this was remarkable by a decrease of the microbial charge and the catalase activity. The results showed that there is a correlation between *Staphylococcus aureus* and the snail mortality rate which confirmed that it is an agent responsible for the higher rates of mortality during raising. Also, snails fed with percentages of rosemary in flour had highest protein amount contrary to sage and peppermint and that positively affected the specific catalase activity. Besides bioactive molecules (phenolic compounds and essential oil), mineral composition of the used plants influenced their activity. The high concentration of minerals is responsible for cell and protein damages and that appeared in the lowest protein amount in both sage and peppermint.

## Figures and Tables

**Table 1:** Mineral content in powdered rosemary, salvia, and peppermint (mg/g of plant powder).

	Rosemary	Sage	Peppermint
Al	0.067	0.127	0.111
Ba	0.006	0.007	0.007
Ca	1.341	2.798	3.534
Fe	0.039	0.094	0.078
K	0.621	0.420	2.351
Mg	0.970	1.955	1.945
Mn	0.008	0.010	0.014
Na	0.764	0.543	1.305
P	0.097	0.139	0.412
Sr	0.004	0.000	0.017
Zn	0.039	0.022	0.021

## Data Availability

No data were used to support this study.
